# Efficacy of Fu’s Subcutaneous Needling for chronic non-specific neck pain and its effect on muscle elasticity: a randomized controlled trial

**DOI:** 10.3389/fmed.2025.1701076

**Published:** 2025-11-11

**Authors:** Zhilin Gu, Ting Zhou, Chunyan Liu, Jiebin Zhu, Yingying Liao

**Affiliations:** 1 The Second Affiliated Hospital of Guangzhou University of Chinese Medicine, Guangzhou, China; 2 Zhongshan Torch Development Zone People's Hospital, Zhongshan, China

**Keywords:** Fu’s Subcutaneous Needling, chronic non-specific neck pain, muscle elasticity, shear wave elastography, randomized controlled trial, standard acupuncture

## Abstract

**Objective:**

Chronic non-specific neck pain (CNNP) is a prevalent cause of disability worldwide, yet effective and safe treatment options remain limited. This study aimed to evaluate the clinical efficacy of Fu’s Subcutaneous Needling (FSN) therapy compared with standard acupuncture and to explore its effect on muscle elasticity using shear wave elastography (SWE).

**Methods:**

In this randomized controlled trial, 70 patients with CNNP were assigned to receive either FSN therapy (*n* = 35) or filiform needle acupuncture (*n* = 35). Clinical outcomes were assessed using the Neck Disability Index (NDI) and Visual Analogue Scale (VAS) before and after treatment and at one-month follow-up. SWE was used to quantify the elasticity modulus of the upper trapezius muscle.

**Results:**

Both groups showed significant improvement after treatment; however, the FSN group demonstrated greater reductions in NDI and VAS scores (*p* < 0.05). SWE values decreased significantly following FSN therapy (*p* < 0.05) but not after filiform acupuncture. Moreover, SWE values correlated strongly with NDI and VAS scores (*p* < 0.05). No adverse events were reported.

**Conclusion:**

FSN therapy is a safe and minimally invasive treatment that provides a relatively good improvement in pain and disability compared with standard acupuncture, while simultaneously restoring muscle elasticity. The integration of SWE as an objective biomarker highlights both the clinical effectiveness and mechanistic insights of FSN, supporting its role as a relatively effective non-pharmacological intervention for CNNP.

**Clinical trial registration:**

Identifier ChiCTR2200062436.

## 1 Introduction

Chronic nonspecific neck pain (CNNP) (1) is defined as pain in the neck and shoulders that cannot be attributed to trauma, infection, tumors, or disease in other parts of the body. Imaging studies of patients experiencing CNNP may reveal degenerative changes in the cervical spine, such as straightening of the physiological curve and osteoporosis. Clinically, CNNP often manifests as persistent pain, s limited range of motion and stiffness in the soft tissues of the neck and shoulders within the anatomical region of the dorsal spine (between the imaginary line of the T1 spinous process and the superior nuchal line) for more than 3 months. Clinical observations indicate that abnormal neck postures, particularly prolonged head-down positions, serve as primary initiating or exacerbating factors for patients with CNNP (2). As a common chronic disease (1), the mean global prevalence of CNNP has reached 24.5% and is increasing on an annual basis (3).

Previous studies have shown that approximately 50 to 85% of patients experience recurrent or persistent pain and other types of discomfort (4). With changes in lifestyle and work patterns, the incidence of CNNP is increasing on an annual basis and is beginning to affect younger people. As a leading cause of disability (1), CNNP has become a significant global public health issue (3) and a major socio-economic burden (5, 6). Despite a wide range of treatments for CNNP, there is currently no agreed standard (1, 7, 8). Drugs are the most commonly used treatment for CNNP in modern medicine, and non-steroidal anti-inflammatory drug (NSAIDs) in particular have demonstrated clinical efficacy for the treatment of pain, especially musculoskeletal and joint pain (1, 7, 8). However, adverse gastrointestinal reactions, kidney damage, and even increased cardiovascular risk are common side effects of NSAID use (9, 10). Acupuncture, particularly filiform needle (FN) therapy, widely applied in clinical practice; some RCTs and systematic reviews suggest potential efficacy, though findings remain heterogeneous (10–12), and its efficacy and safety for neck pain have been demonstrated (13–15).

Fu’s Subcutaneous Needling (FSN) therapy exhibits unique advantages for the treatment of musculoskeletal pain, particularly by virtue of its ability to rapidly relieve pain and improve limb function (16). Unlike traditional acupuncture, FSN does not rely on predefined acupoints or the traditional concept of the “Deqi” sensation (historically described as soreness, numbness, distension, or heaviness after needle insertion in Traditional Chinese Medicine (TCM)) (17). The stimulation of tension muscles associated with pain (defined as “affected muscles” in floating needle therapy) is commonly observed in most patients with neck pain (18–20). Instead, FSN is performed along the subcutaneous tissue with fewer insertions and is considered minimally invasive, which may enhance patient comfort. Past treatments and research have focused solely on alleviating pain symptoms in patients with chronic neck pain, while neglecting the tension and stiffness within the muscular system. This may also be a contributing factor to the recurrent nature of chronic neck pain (3). Ischemic injury to muscles and fascia is a common cause of pain (21, 22). Clinical practice has shown that floating needle therapy, through sweeping and reperfusion techniques that directly stimulate superficial fascia and muscle tissue, can effectively improve local circulation and energy metabolism in damaged muscles (14, 23–25). This alleviates muscle tension and reduces pain. However, there is currently a lack of objective evaluation metrics to substantiate the efficacy of floating needle therapy in improving muscle hardness. The role of SWE as an objective, non-invasive metric for assessing skeletal muscle elasticity has also been confirmed (26, 27). Therefore, this study employed a randomized controlled trial design. Through objective evaluation using SWE and in conjunction with scales such as the NDI, and using the widely recognized FN protocol as a control, it further confirmed the efficacy of floating needle therapy in alleviating pain, improving cervical spine function, and relieving muscle tension in patients with chronic neck pain.

## 2 Methods

### 2.1 Trial registration

This study was prospectively registered at the Chinese Clinical Trial Registry (ChiCTR) under the identifier ChiCTR2200062436 on 07 August 2022. The registered title was “Clinical study of FSN for cervical spondylosis-related neck pain based on shear wave elastography (SWE).” For the purpose of international readership and in accordance with our diagnostic criteria, the condition is referred to in this manuscript as CNNP, which corresponds to the same patient population defined in the registration.

### 2.2 Clinical data

#### 2.2.1 General information

Seventy-two patients with CNNP were recruited as study subjects from Guangdong Provincial Hospital of Traditional Chinese Medicine between July 2022 and July 2023. The 72 patients were randomly divided into a FSN group (36 cases) and the FN as the control group (36 cases) using a random number table. The randomization sequence was generated by an independent individual using a computer-generated list of random numbers, which were then transferred into consecutively numbered, opaque, sealed envelopes. Each envelope contained a card indicating the group assignment based on the pre-generated sequence. Eligible participants were assigned to groups according to the information on the card after signing the informed consent form. Due to the specificity of acupuncture treatment, double-blinding could not be implemented; therefore, blinding was only applied for efficacy evaluation and data analysis personnel. This research was approved by the Ethics Committee of Guangdong Provincial Hospital of Chinese Medicine (Ethics Number: BF2022-017-01, approved on February 25, 2022).

#### 2.2.2 Diagnostic criteria

The diagnostic criteria for CNNPP were based on previous guidelines (28), including (1) a predominant complaint of abnormal sensations such as pain in the occiput, neck, and shoulder, which may be accompanied by tenderness in the corresponding areas; (2) persistent pain for more than 3 months; and (3) degenerative changes in the cervical vertebrae, as determined by imaging examinations.

#### 2.2.3 Inclusion criteria

The inclusion criteria were as follows: (1) met the diagnostic criteria for CNNP; (2) predominant complaint of neck and shoulder pain; (3) aged between 18 and 75 years; (4) palpate the upper trapezius muscle along its direction of fiber orientation to detect muscle tension and stiffness or the presence of MTrP within the muscle, which contain one or more MTrPs; (5) a Visual Analogue Scale (VAS) score between 3 and 8, and (6) subjects volunteered to participate in this study and provided signed consent.

#### 2.2.4 Exclusion criteria

The exclusion criteria were as follows: (1) patients with severe diseases of other systems, who were considered unsuitable for participation by the researchers; (2) neck pain caused by other diseases such as scapulohumeral periarthritis and myofascitis; (3) patients who had undergone cervical surgery, or those with severe congenital cervical deformities; (4) patients who refused acupuncture or had contraindications to acupuncture; (5) patients with damaged skin, severe rash, or other conditions on the neck and shoulders that were not suitable for ultrasonography, and (6) patients receiving concomitant therapy such as analgesics or physiotherapy.

#### 2.2.5 Elimination and withdrawal criteria

Patients were eliminated or could withdraw in accordance to the following conditions: (1) poor compliance or unable to complete the treatment according to the prescribed course, and (2) self-withdrawal from the study.

#### 2.2.6 Termination criteria

The researchers were able to terminate the inclusion of certain patients if serious adverse events occurred or complications became evident during the trial.

### 2.3 Therapeutic methods

#### 2.3.1 The FSN group

**Figure 1:**
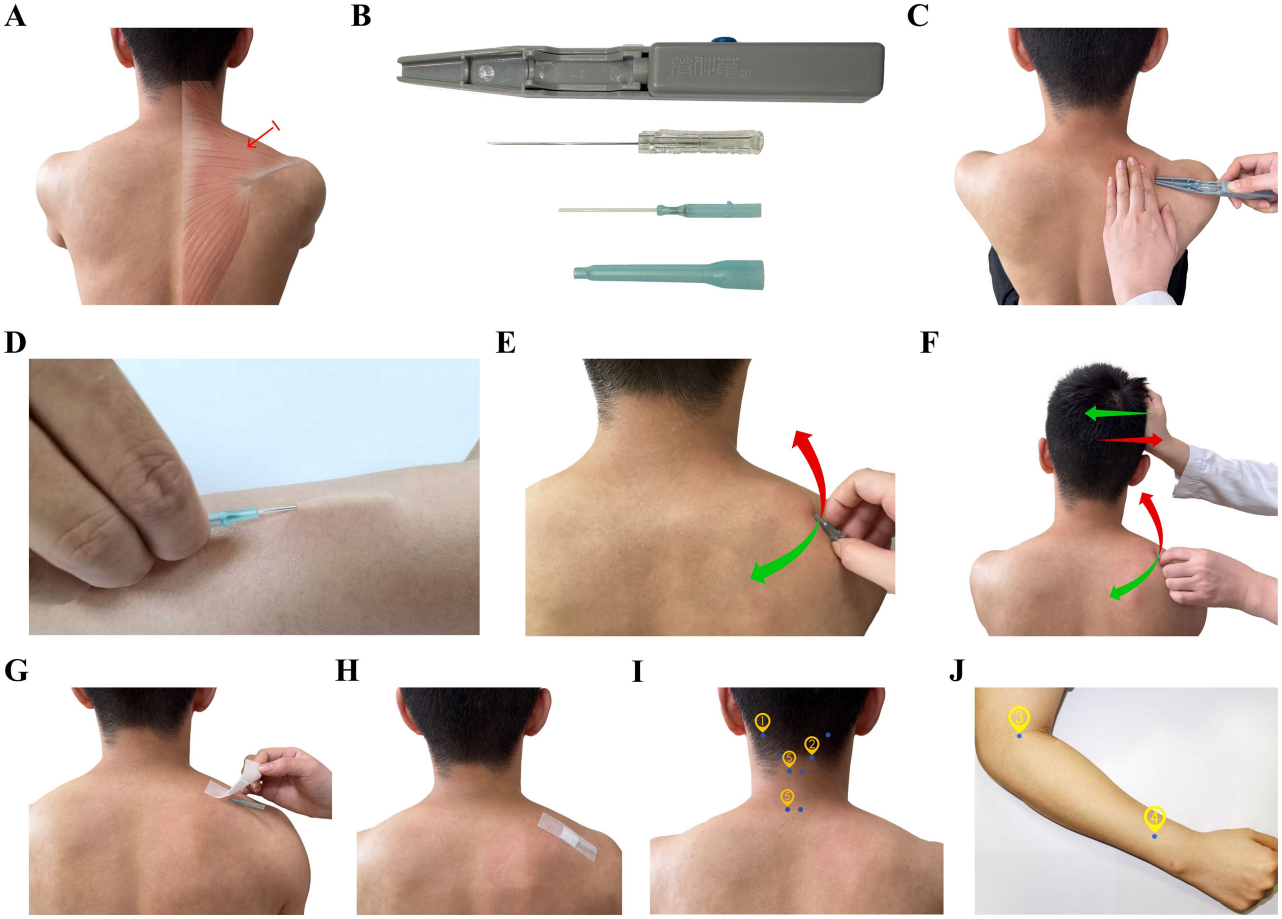
Schematic diagram of the therapeutic methods. **(A)** Acupuncture point and targeted muscle; **(B)** Fu’s Subcutaneous needle and insertion device; **(C)** Image showing how the Fu’s Subcutaneous Needling Therapy needle was inserted into the skin; **(D)** Image showing how the needle was pushed slowly along the loose connective tissue under the skin; **(E)** Image depicting the sweeping movement; **(F)** Image showing the reperfusion approach for the superior trapezius muscle; **(G,H)** Image showing how the soft casing was retained; **(I,J)** Images showing the acupoints used in the filiform needle group.

The treatment procedure for Fu’s Subcutaneous Needling follows established protocols described in the relevant professional literature (29). Based on preliminary clinical observations, the upper trapezius muscle (Figure 1A) was identified as one of the most common “affected muscles” in patients with CNNP. Considering the technical characteristics of elastic ultrasound, this study was limited to the inclusion of patients with the superficial muscle, specifically the upper trapezius as the “affected muscle” (Figure 1A). The needle was inserted at the Jianjing acupoint (GB 21, Figure 1A) on the affected side (the midpoint of the line connecting the spinous process of the seventh cervical vertebra and the acromion). Following routine disinfection, a disposable M-size FSN needle (Nanjing Paifu Medical Science and Technology Co., Nanjing, China) was inserted into the subcutaneous tissue with the needle tip pointing in the direction of the spine using a specialized needle inserter (Figures 1B,C). The needle was then slowly advanced along the loose connective tissue under the skin to a depth of approximately 30 mm while holding the needle seat (Figure 1D). The needle core was then withdrawn into the casing, and a fan-shaped scattering action was performed for 2 min at a frequency of approximately 100 times per minute, with a scattering range of approximately 40° to 45° by using the thumb as a fulcrum, keeping the index and ring fingers swinging softly and smoothly (Figure 1E). During the swaying movement, we performed FSN muscle reperfusion techniques, instructing the patient to perform ipsilateral shoulder shrugging and side-to-side head movements while providing equal resistance (Figure 1F). Each reperfusion action lasted for approximately 10 s and was repeated twice. After the patient’s neck and shoulder pain and other symptoms had been significantly relieved or disappeared, and the tightness, stiffness, hardness, and slipperiness of the affected muscle under palpation disappeared, the needle core was withdrawn. The soft casing was left under the skin and fixed with medical adhesive tape for 6 h before removal (Figures 1G,H). All operators possess over 5 years of clinical experience and have undergone standardized operational training prior to trial implementation.

#### 2.3.2 The FN group

FN treatment was conducted, as described previously (30, 31) using several selected acupoints: Tianzhu (BL10), Fengchi (GB20), Quchi (LI11), Waiguan (TE5), Jingjiaji (C3 and C7), and Ashi points (Figures 1I,J; Table 1), the description of acupoints such as BL10 follows TCM nomenclature; these terms are traditional and do not have direct biomedical equivalents, in this trial, the anatomical landmarks were standardized to ensure reproducibility across practitioners. Following routine disinfection, we used disposable sterile acupuncture needles (0.35 × 25 mm; Suzhou Tianyi Acupuncture and Moxibustion Apparatus Co., Ltd.). The needles were retained for 30 min after achieving the traditionally described sensation of “Deqi (as characterized by feelings of soreness, numbness, distension, and heaviness).”

**Table 1:** Acupoints used in the filiform needle group.

Acupoints	Position	Needle insertion
① Fengchi (GB 20)	In the neck, under the occipital bone, flush with Fengfu acupoint, in the depression between sternocleidomastoid muscle and the upper end of trapezius muscle.	Oblique needle insertion toward to angle of mandible at depth of 15–20 mm.
② Tianzhu (BL 10)	In the neck, at the hairline depression behind the outer edge of trapezius muscle, approximately 1.3 inches apart from the middle of the hairline.	Perpendicular needle insertion at depth of 12–25 mm.
③ Quchi (LI 11)	At the lateral end of elbow striation, bend your elbow, the midpoint of the line between Chize point and lateral epicondyle of humerus.	Perpendicular needle insertion at depth of 15–30 mm.
④ Waiguan (SJ 5)	At the back of forearm, on the connecting line between Yangchi point and elbow tip, 2 cun upper the horizontal stripes of wrist back, between ulna and radius.	Perpendicular needle insertion at depth of 15–20 mm.
⑤ Jiaji of the cervical spine (EX-B 2)	In the neck, under the spinous processes of the third and seventh cervical vertebrae, 0.5 cun lateral.	Oblique needle insertion toward to vertebral column at depth of 15–20 mm.

#### 2.3.3 Treatment course

Treatment was administered three times per week for a total of six treatments over 2 weeks.

### 2.4 Observation of therapeutic effects

#### 2.4.1 The neck disability index

The Neck Disability Index (NDI) (32) comprises assessments in two parts across 10 dimensions: neck pain and related symptoms (including pain level, headache, concentration, and sleep), as well as daily life activities and functional abilities (such as personal care, lifting heavy objects, reading, work, driving, and entertainment). The NDI is the most commonly used method for reporting outcomes in patients with neck pain (33). Patients self-evaluate each item on a scale of 0 to 5 based on severity, with higher scores indicating more severe functional impairment. The NDI (%) was calculated as follows: (total score of all items) / (number of items completed by the subject × 5) × 100%.

#### 2.4.2 Visual analogue score for pain

The VAS for pain (31) is the most commonly used uni-dimensional assessment tool for pain intensity with a range of advantages, including accuracy, high sensitivity, simplicity, and ease of use. As a self-reporting tool, the VAS has been recognized as the ‘gold standard’ for pain assessment (34). The VAS involves asking the patient to mark a point on a 10 cm line with 10 marked intervals, representing varying degrees of pain. The line starts at “0” (representing no pain) and ends at “10” (representing the most severe imaginable pain). Patients mark the corresponding value based on their immediate level of pain: 0 indicates no pain, 1–3 indicates mild pain, 4–6 indicates moderate pain, 7–9 indicates severe pain, and 10 indicates extreme pain.

#### 2.4.3 SWE

A Supersonic Aixplorer color Doppler ultrasound diagnostic instrument (Produced by SuperSonic Imagine, Aix, France) was used, with an L10-2 linear array probe selected, operating at a frequency of 2–10 MHz in musculoskeletal ultrasound examination mode. Patients were positioned prone, with their neck and shoulders fully relaxed. The measurement point was set at the midpoint of the line connecting the acromion and the spinous process of the seventh cervical vertebra. Muscle echoes and fiber orientation were observed using two-dimensional ultrasound, with the sound beam perpendicular to the muscle. After switching to SWE mode and obtaining an ideal image, the image was frozen, and the Q-BOX function was activated to measure the Young’s modulus (E) and shear wave velocity (SWV) of the muscle within a specific region of interest (ROI). The ROI was set as a circle with a diameter of 5 mm. All parameters were independently measured three times by the same experienced senior attending physician in the ultrasound department and then averaged for analysis. All operators possess over 5 years of clinical experience and have undergone standardized operational training prior to trial implementation.

### 2.5 Safety evaluation

Adverse reactions, such as needle sickness, subcutaneous hematoma, broken needles, and needle retention during the trial, were all recorded.

### 2.6 Statistical analysis

Statistical analysis was conducted by an independent third party to avoid bias. All data were entered using WPSEXCEL (Beijing Kingsoft Office Software Company Limited, Beijing, China) software and analyzed using SPSS version 26.0 (IBM Corporation, Armonk, New York, USA) statistical software. Cases that were excluded or withdrew were not included in the final analysis. Measurement data are expressed as mean ± standard deviation. If the data followed a normal distribution, as determined by the Shapiro–Wilk test, we used a t-test for statistical comparison; if not, a rank sum test was applied. Additionally, non-parametric tests were used for non-normally distributed data. The Mann–Whitney U test was applied to two independent samples, while the Wilcoxon signed-rank test was used for paired samples. Count data and ordinal data are expressed as proportions (%). For count data in a unidirectional ordered R × C table, we used the rank sum test to compare the differences in effects among treatment groups, and the rank sum test was used for ordinal data. Correlations between SWE and NDI / VAS scores were analyzed using Pearson correlation analysis. All statistical tests were two-sided, *p* < 0.05 was considered statistically significant.

## 3 Results

### 3.1 Patient recruitment and normality testing

**Figure 2:**
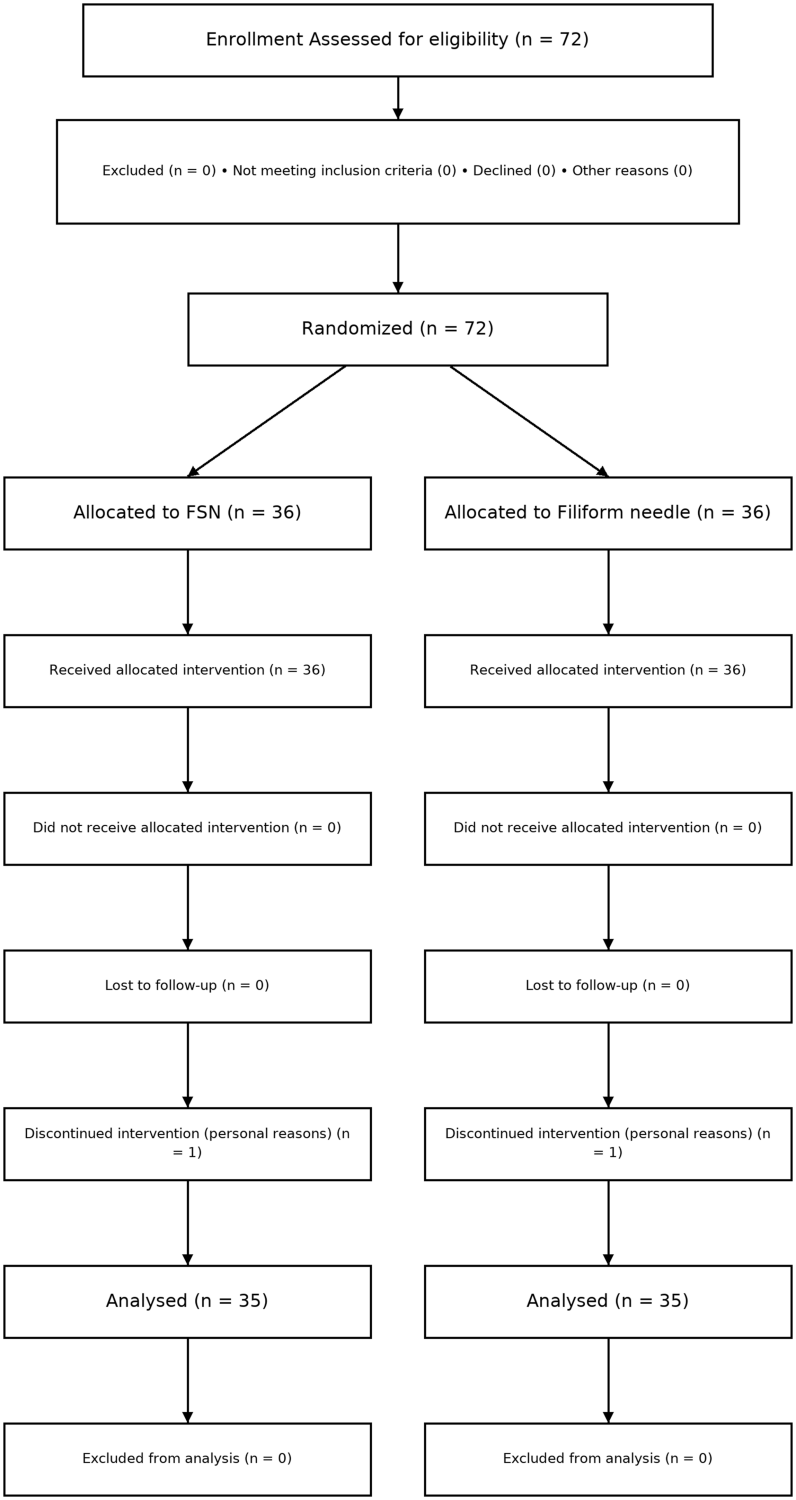
CONSORT flow diagram of patient enrollment, allocation, follow-up, and analysis.

The patient enrollment and allocation are shown in Figure 2 (CONSORT flow diagram). During the treatment process, one patient from each group withdrew due to personal reasons, resulting in a total of 70 patients being included in the final analysis. There were 35 patients in the FSN group and 35 patients in the FN group. Normality testing showed that with the exception of post-treatment SWE (W = 0.969, *p* = 0.079), which exhibited a normal distribution (*p* > 0.05), all other variables did not conform to a normal distribution (*p* < 0.05). See Table 2 for details.

**Table 2:** Results of the normality test (Shapiro–Wilk test).

Variable	*n*	W	*p*
Age (years)	35	0.957	**0.016**
Total Course (years)	35	0.9	**0**
Daily Head-Down Time (hours)	35	0.961	**0.03**
NDI score before treatment	35	0.934	**0.001**
NDI score after treatment	35	0.903	**0**
NDI score for follow-up	35	0.918	**0**
Effective rate	35	0.943	**0.003**
VAS score before treatment	35	0.912	**0**
VAS score after treatment	35	0.945	**0.004**
VAS score for follow-up	35	0.914	**0**
The SWE before treatment	35	0.834	**0**
The SWE after treatment	35	0.969	0.079

Bold values indicate significant differences.

### 3.2 Group comparisons

There were no statistically significant differences between the two groups in terms of age, total course of disease, and daily head-down time (*p* > 0.05), thus indicating comparability (Table 3).

**Table 3:** A comparison of general information for CNNP patients in the two groups.

Group	Age (years)	Total course (years)	Daily Head-Down Time (hours)
FSN group	36.00 (27.00, 54.00)	5.67 (2.17, 8.00)	6.00 (5.00, 8.00)
FN group	51.00 (37.00, 53.00)	5.25 (3.75, 7.92)	6.00 (5.00, 8.00)
Z	1.870	0.587	0.006
*P*	0.620	0.557	0.995

### 3.3 Comparison of NDI scores before and after treatment and during follow-up

The median difference between the FSN group and the FN group at after treatment and follow-up, along with their respective 95% confidence intervals, were 12.0 (9.33–15.56) and 11.78 (9.11–14.00). Before treatment, there was no statistically significant difference in NDI scores (%) between the two groups (*p* > 0.05), thus indicating comparability. However, after treatment and during follow-up, the NDI scores (%) of both groups decreased when compared to those before treatment (*p* < 0.05). Furthermore, the NDI scores of the FSN group were lower than those of the FN group at both evaluation time points (*p* < 0.05) (Table 4).

**Table 4:** Comparison of neck disability index (NDI) scores (%) between the two groups before and after treatment and during follow-up.

Group	Before treatment	After treatment	Follow-up
FSN group	34.00 (28.00, 42.22)	4.44 (2.22, 8.00)^a^	4.00 (2.00, 6.67)^b^
FN group	36.00 (26.67, 42.00)	16.00 (13.33, 24.00)^c^	14.00 (11.11, 20.00)^d^
Z	0.141	6.332	6.622
*p*	0.888	**< 0.001**	**< 0.001**

Compared with the same group before treatment: ^a^ (*Z*: 5.162, *p* < 0.001); ^b^ (*Z*: 5.161, *p* < 0.001); ^c^ (*Z*: 5.144, *p* < 0.001); ^d^ (*Z*: 5.088, *p* < 0.001). Bold values indicate significant differences.

### 3.4 Comparison of VAS scores before and after treatment and during follow-up

The median difference between the FSN group and the FN group at after treatment and follow-up, along with their respective 95% confidence intervals, were 2 (2–3) and 2 (2–2). There was no statistically significant difference in VAS scores between the two groups before treatment (*p* > 0.05), thus indicating comparability. After treatment and during follow-up, the VAS scores of both groups decreased compared to those before treatment (*p* < 0.05), and the VAS scores of the FSN group were lower than those of the FN group (*p* < 0.05) (Table 5).

**Table 5:** Comparison of VAS scores before and after treatment and during follow-up between the two groups.

Group	Before treatment	After treatment	Follow-up
FSN group	7.00 (5.00, 7.00)	1.00 (1.00, 2.00)^a^	1.00 (1.00, 1.00)^b^
FN group	6.00 (5.00, 7.00)	4.00 (3.00, 5.00)^c^	3.00 (2.00, 4.00)^d^
Z	0.850	5.727	6.441
*p*	0.395	**< 0.001**	**< 0.001**

Compared with the same group before treatment: ^a^ (*Z*: 5.194, *p* < 0.001); ^b^ (*Z*: 5.203, *p* < 0.001); ^c^ (*Z*: 5.307, *p* < 0.001); ^d^ (*Z*: 5.208, *p* < 0.001). Bold values indicate significant differences.

### 3.5 Comparison of SWE of the upper trapezius muscle before and after treatment

**Figure 3:**
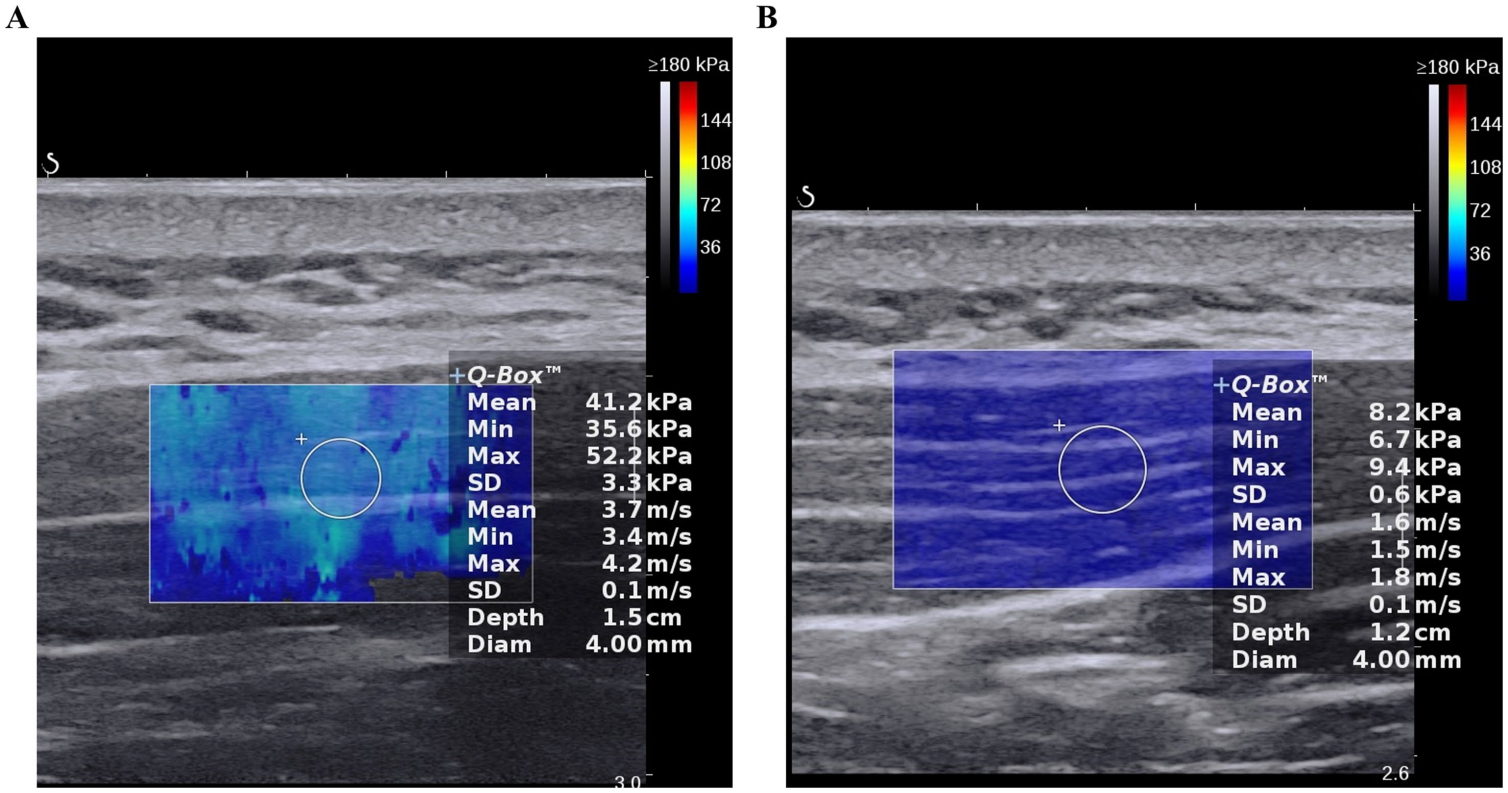
The SWE elastic modulus value prior to treatment and following treatment in Fu’s Subcutaneous Needling (FSN) Group; **(A)**. Prior to treatment; **(B)**. Following treatment.

The median difference between the FSN group and the FN group at after treatment, along with their respective 95% confidence intervals, were 2.167 (0.667–3.367). Before treatment, there was no statistically significant difference in the SWE elastic modulus values of the upper trapezius muscle between the two groups (*p* > 0.05), thus indicating comparability. After treatment, the SWE elastic modulus values of the upper trapezius muscle in the FSN group decreased significantly compared with that before treatment (*p* < 0.05, Figures 3A,B); there was no such reduction in the FN group (*p* > 0.05) (Table 6).

**Table 6:** Comparison of mean SWE elastic modulus values of the upper trapezius muscle before and after treatment in the two groups.

Group	Before treatment	After treatment	*Z*	*p*
FSN group	14.07 (10.57, 17.10)	9.17 (8.17, 11.33)	4.513	**< 0.001**
FN group	12.10 (10.17, 15.67)	11.77 (9.90, 14.00)	1.785	0.074
*Z*	1.304	2.849		
*p*	0.192	**0.004**		

Bold values indicate significant differences.

### 3.6 Correlation analysis between VAS score, NDI score, and SWE elastic Modulus

Spearman’s correlation analysis revealed a strong positive correlation between VAS score, NDI score, and SWE elastic modulus values (Table 7).

**Table 7:** Correlation analysis between VAS score, NDI score, and SWE elastic modulus values after treatment.

Statistical value	VAS score	NDI score
*r*	0.407	0.347
*p*	**< 0.001**	**0.003**

Bold values indicate significant differences.

### 3.7 Statistical power analysis

This study employed a *post-hoc* statistical power analysis to assess the test power for detecting a specific effect size given the current sample size. The power analysis was conducted using SPSSAU (The SPSSAU project, 2025), with a significance level (*α*) set at 0.05 (two-tailed) and a sample size of 35. The statistical power value (1 - Beta value) for this study was 0.841, indicating a strong ability to detect the effect under the current conditions (35).

### 3.8 Safety evaluation

No adverse events were recorded in either group (e.g., needle sickness, bent needles, broken needles, bleeding, or subcutaneous hematoma); thus, the incidence of adverse reactions was 0%.

## 4 Discussion

Within the framework of TCM, CNNP has historically been described as a type of “obstruction syndrome” attributed to impaired circulation and external pathogenic factors. A distinction is made between ‘pain due to obstruction’ and ‘pain due to lack of nourishment, thus reflecting the concepts of deficiency and excess. These concepts are traditional interpretations without direct biomedical equivalents. The pathological mechanisms underlying CNNP remain unclear. However, current hypotheses suggest potential associations with muscle dysfunction, peripheral nociceptor activity, central sensitization, and secondary biomechanical imbalances (35–38).

Clinical observations indicate that abnormal neck postures, particularly prolonged head-down positions, serve as primary initiating or exacerbating factors for patients with CNNP (39). Prolonged abnormal postures can result in excessive fatigue of associated muscles, subsequently altering muscle movement patterns and leading to the deactivation of deep muscles. To preserve neck stability and normal physiological functionality, the superficial muscles compensate to increase their activation to substitute for the function of deep muscles (40, 41), ultimately resulting in increased hardness of the superficial muscles (19–21). This muscular tension triggers the excessive release of acetylcholine, further exacerbating sustained muscle contraction and local ischemia, thereby establishing a vicious cycle of “pain-muscle tension-ischemia-pain” (38, 42). Consequently, the key to treating CNNP lies in disrupting this vicious cycle by alleviating neck muscle tension, relieving muscular ischemia, and restoring mechanical balance. Given the side effects associated with non-steroidal drugs, non-pharmacological treatment options are preferred (1). Of these, correcting structural and mechanical imbalances is a critical component of multimodal therapy (1, 43). The remarkable efficacy and safety of acupuncture have made it a mainstream therapy (9, 13–15).

Unlike conventional acupuncture or pharmacological approaches, FSN does not rely on acupoints or the traditionally described Deqi sensation, requires fewer needle insertions, and is minimally invasive. Compared with NSAIDs, FSN avoids gastrointestinal or cardiovascular risks, highlighting its value as a safe non-pharmacological option. This therapeutic approach uses a disposable floating needle (FSN needle) that is applied subcutaneously to muscle regions clinically identified as tense and pain-related, often referred to in FSN practice as “affected muscles.” These muscles often harbor multiple myofascial trigger points (MTrPs) (29). Related studies have shown that FSN therapy demonstrated more significant clinical efficacy in skeletal muscle-related (24, 44, 45). Previous research demonstrated that over half of patients with CNNP exhibit identifiable MTrPs within their neck musculature (46). Notably, the upper trapezius muscle is the most frequently affected muscle in CNNP patients, with a prevalence of MTrPs reaching 38.5% (36). The integration of SWE as an imaging biomarker is a key strength of this study. By demonstrating a strong correlation between SWE values and clinical scores, our findings support the use of SWE as an objective marker for monitoring therapeutic effects in musculoskeletal pain, which enhances reproducibility and international applicability (18, 26, 27, 47).

Ischemic injury to muscles and fascia is a common cause of pain (21, 22). FSN therapy targets the subcutaneous loose connective tissue that is intricately associated with muscle tissue (48). By employing two techniques, sweeping and reperfusion, FSN therapy aims to eliminate MTrPs and alleviate muscle tension, thereby ameliorating the ischemic and hypoxic conditions of the affected muscle. However, its specific molecular mechanisms remain unclear. During floating needle therapy, the reperfusion activity we employ enables targeted contraction and stretching of the target muscles, thereby increasing local blood flow (49) and improving regional circulation in damaged musculoskeletal tissues. This ultimately alleviates ischemia and hypoxia in MTrPs (14, 23, 24). The sweeping and dispersing activity stretches the subcutaneous fascia layer while simultaneously stimulating the muscle tissue beneath the fascia. This improves muscle energy metabolism and reduces muscle hardness by increasing the content of mitochondrial CS and Complex II, and enhancing the active expression of COX-I protein in muscle tissue (24, 25). This represents a decrease in shear wave elasticity and an increase in muscle elasticity (26). Based on prior research, we propose that the enhancement of muscle elasticity may suppress aberrant spindle activity (50) and modulate both inflammation and endoplasmic reticulum stress (44), leading to pain reduction. Ultimately breaking the vicious cycle of “pain-muscle tension-ischemia-pain.” From the standpoint of TCM theory, loose connective tissue is posited to serve as the material foundation for meridians, a traditional concept without direct biomedical equivalent (51). FSN therapy, which directly targets this specific tissue layer, is purported to rapidly facilitate the unblocking of meridians and collaterals, thereby alleviating pain, although these explanations are based on historical usage rather than contemporary scientific validation. When practicing FSN therapy, identifying the “affected muscles,” both pre- and post-treatment, predominantly depends on the physician’s palpation, thus introducing a degree of subjectivity.

In this study, we incorporated the use of musculoskeletal ultrasound to objectively assess the “hardness” of the affected muscles and to evaluate the efficacy of floating needle therapy in the treatment of CNNP, thereby enhancing the scientific rigor of our assessment. FSN devices have been included in the National Medical Products Administration medical device catalogue and have been evaluated in studies consistent with Food and Drug Administration safety standards (52). With these certifications, FSN has been widely applied in clinical practice, and accumulating evidence has demonstrated its significant therapeutic efficacy.

This study has some limitations that need to be considered, such as a relatively small sample size, short follow-up period and a lack of more objective indicators to explore its mechanism. Given the small sample size, future studies should employ larger samples to investigate the impact of gender and age-related factors. This study did not employ an intention-to-treat (ITT) analysis. Furthermore, without including a sham or waiting-list control, making it difficult to distinguish specific FSN effects from non-specific placebo effects. Future studies should include sham controls or multi-arm designs, integrate magnetic resonance imaging and other imaging modalities to comprehensively assess the SWE characteristics of multiple neck muscles. Future research should analyze the differential responses of various muscles to floating needle therapy, investigate the potential mechanisms by which this therapy ameliorates CNNP, such as improvements in muscle microcirculation and alterations in inflammatory factors, and investigate the correlation between these mechanisms and SWE outcomes.

## 5 Conclusion

The findings of this study demonstrate that FSN therapy effectively reduces NDI and VAS scores in patients with CNNP and enhances muscle elasticity, and suggests a strong positive correlation indicating that this therapy primarily alleviates muscle tension to relieve pain and improve cervical spine function. Therefore, we can use SWE to determine the tense muscles (affected muscles) associated with pain more accurately, in order to achieve better therapeutic results. Furthermore, FSN therapy involves fewer needle insertions and does not depend on the “deqi” sensation, as characterized by feelings of soreness, numbness, distension, and heaviness; these symptoms are commonly associated with traditional acupuncture. This procedure is almost “painless” due to the minimal discomfort associated with needle insertion, thus enhancing patient acceptance and compliance.

## Data Availability

The raw data supporting the conclusions of this article will be made available by the authors, without undue reservation.
